# Current evidence for J147 as a potential therapeutic agent in nervous system disease: a narrative review

**DOI:** 10.1186/s12883-023-03358-5

**Published:** 2023-09-06

**Authors:** Fang Qiu, Yanmei Wang, Yunbo Du, Changchun Zeng, Yuqiang Liu, Haobo Pan, Changneng Ke

**Affiliations:** 1 Department of Burn and Plastic Surgery, Shenzhen Longhua District Central Hospital, Shenzhen, 518110 Guangdong China; 2 Center for Human Tissues and Organs Degeneration, Shenzhen Institutes of Advanced Technology, Chinese Academy of Sciences, Shenzhen, 518055 Guangdong China; 3 Department of critical care medicine, Shenzhen Longhua District Central Hospital, Shenzhen, Guangdong China; 4 Department of Medical Laboratory, Shenzhen Longhua District Central Hospital, Affiliated Central Hospital of Shenzhen Longhua District, Guangdong Medical University, Shenzhen, Guangdong China; 5 Department of Anesthesiology, Shenzhen Second People’s Hospital, The First Affiliated Hospital of Shenzhen University, Shenzhen, 518025 Guangdong China

**Keywords:** J147, Neuroprotective, Nervous system disease, Drug, Therapeutic, Review

## Abstract

Curcumin has anti-inflammatory, antioxidant, and anticancer effects and is used to treat diseases such as dermatological diseases, infection, stress, depression, and anxiety. J147, an analogue of curcumin, is designed and synthesized with better stability and bioavailability. Accumulating evidence demonstrates the potential role of J147 in the prevention and treatment of Alzheimer’s disease, diabetic neuropathy, ischemic stroke, depression, anxiety, and fatty liver disease. In this narrative review, we summarized the background and biochemical properties of J147 and discussed the role and mechanism of J147 in different diseases. Overall, the mechanical attributes of J147 connote it as a potential target for the prevention and treatment of neurological diseases.

## Introduction

### Discovery and characteristics of J147

Turmeric (Curcuma longa) belongs to the ginger family (Zingiberaceae), and primarily flourishes in Malesia, India, China, Polynesia, and Thailand [1]. Turmeric is widely used in traditional Indian Ayurvedic medicine, traditional Chinese medicine, and Southeast Asian medicines. The therapeutic effect of Turmeric’s main active ingredient is curcumin, which has anti-inflammatory [2], antioxidant [2], and anticancer activities [3]. In addition to treating dermatological diseases and infections, curcumin has also been used to relieve stress and depression, presumably through increasing the serotonin and dopamine concentrations in the central nervous system (CNS) as well as inhibiting monoamine oxidase (MAO) activity [4, 5]. Moreover, curcumin has been demonstrated to reverse cognitive dysfunction in animal models of Alzheimer’s disease (AD) (e.g., Tg2576, APPswe/PS1dE9, 3xTg-AD mice and 22 month SD rats) suggesting its potential neurorestorative effects [6, 7].

Unfortunately, despite its safety at high doses, curcumin has low bioavailability and selectivity [8]. Curcumin is poorly absorbed, rapidly metabolized, and systemically eliminated: the oral bioavailability of curcumin in rats is less than 1%, with an elimination half-life (t_1/2_) of less than 5 min [9]. In addition, curcumin cannot effectively penetrate the blood-brain barrier (BBB); thus, additional techniques, such as nanocarriers, are required to improve its BBB permeation [10]. Although in vitro studies have demonstrated curcumin’s neuroprotective activities [11], such as inhibiting amyloid β (Aβ) production by targeting β-secretase (BACE1) [12], the assessment of curcumin’s function in the CNS is hampered in vivo [10].

**Fig. 1:**
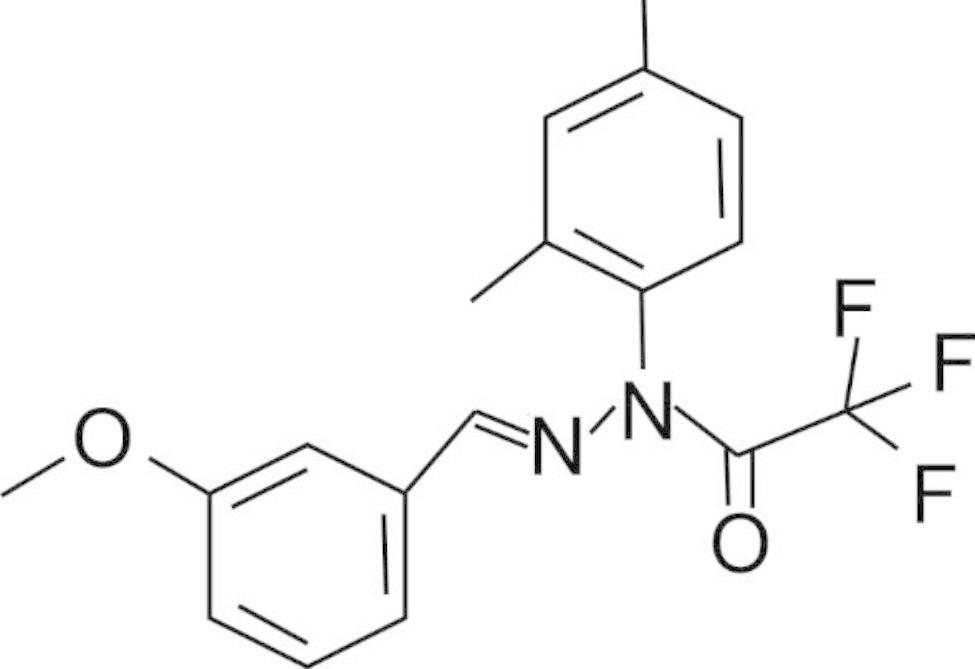
Chemical structure of J147 [14]. The structure of J147 originated from the hypothetically bioactive fragment of CNB-001 (not shown) [14], with the central pyrazole ring partly replaced by a trifluoroacetamide group, which reduced the molecular weight of CNB-001 and potentially increased its solubility

In order to improve the pharmacodynamic and pharmacokinetic properties of curcumin, Liu et al. synthesized a series of hybrid molecules between curcumin and cyclohexyl-bisphenol A, a compound with neuroprotective and neurotrophic activities [13]. One of the hybrid compounds, CNB-001, stood out with superior stability and neuroprotective abilities over curcumin in multiple neurotoxicity assays. Later, Chen et al. found a compound with higher potency called J147 among many CNB-001 derivatives [14]. The chemical structure of J147 is presented in Fig. 1.

J147 conforms to Lipinski’s rule of five (RO5), a set of guidelines to evaluate the potential of a compound to become a drug [15]. According to RO5, a good drug candidate should have a molecular weight of < 500 Dalton, a partition coefficient (cLogP) < 5, no more than five hydrogen bond donors, and no more than ten hydrogen bond acceptors. J147 has a relatively low molecular weight of 351 Dalton, a cLogP of 4.5, and a total polar surface area of 41.9 [16, 17] and is, in theory, a good CNS drug candidate. In preliminary tests, J147 exhibited broad neuroprotective effects both in vitro and in vivo [14]. Specifically, J147 could rescue embryonic cortical cell death from trophic factor withdrawal at a low concentration (EC_50_ = 25 nM) [14]. J147 also showed brain-derived neurotrophic factor (BDNF)-like activity, anti-oxidative stress activity, and anti-amyloid toxicity activity in different cell lines at concentrations between 10 nM and 200 nM, far overtaking curcumin, which was mostly inactive in these tests at the same concentrations [14]. In animal experiments, J147 has been shown to enhance the long-term potentiation and memory in healthy Sprague-Dawley rats and C57BL6/J mice as evidenced by behavioral experiments, including novel object recognition tests, Barnes mazes, and Y mazes [14]. Moreover, orally administered J147 prevented memory deficits in the APPswe/PS1/∆E9 transgenic AD mouse model, presumably through reducing the soluble amyloid beta (Aβ) levels, as well as reducing oxidative stress and inflammatory response in the hippocampus [14]. Subsequent studies revealed various other functions of J147 in the brain, including decreasing fatty acid levels [18, 19], protecting blood-brain barrier permeability hemostasis [20], improving brain vascular function [20], and enhancing the production of new brain cells [21]. These facts suggested the remarkable neuroprotective activity of J147 and emphasized its strong potential in AD therapy.

Besides neurotrophic and neuroprotective effects, J147 has demonstrated appreciable bioavailability and safety over curcumin [22]. The oral bioavailability and t_1/2_ of J147 in mice are 28% and 2.5 h, respectively [23, 24]. Despite being an acylhydrazine, a group of compounds that may be metabolized into carcinogenic or toxic aromatic amines or hydrazines, a detailed analysis was performed to explore the metabolic products of J147 using mice and human microsomes [23]. In this study, it was revealed that the hydrazone scaffold of J147 remained intact, and no hydrolysis of the amide or trifluoroacetamide was observed. A total of five metabolites were identified, which were simple oxidation products of J147, and none of them showed potential hazardous effects. Some metabolites even showed similar neuroprotective activities to J147. Furthermore, the presence of sulfonated or glucuronidated metabolites suggested that J147 was unlikely to induce significant metabolic toxicity. Hence, J147 has a promising safety and stability profile that positions it as a strong candidate for further drug development studies.

The intracellular target of J147 was first identified in 2018 by Joshua Goldberg et al. to be the mitochondrial α-F1-ATP synthase (ATP5A) in vitro and in vivo [25]. By inhibiting ATP5A, J147 induces an increase in the cytosolic Ca^2+^ concentration in a dose-dependent manner. The elevated Ca^2+^ leads to the activation of calcium/calmodulin-dependent protein kinase kinase β (CAMKK2), which then activates the AMPK/mTOR pathway, a canonical aging- and dementia-related signaling pathway that is known to attenuate age-associated decline and extend lifespan [26]. A later study also revealed that by modulating the AMPK pathway, J147 causes the inhibition of acetyl-CoA carboxylase 1 (ACC1), an enzyme responsible for converting acetyl-CoA into malonyl-CoA, the precursor of free fatty acids (FFAs) [19]. This may explain the effect of J147 in reducing plasma and brain fatty acid levels.

Accumulating evidence suggests that J147 may have therapeutic effects not only for AD, but also for diabetic neuropathy [27], ischemic stroke (IS) [28], traumatic brain injury (TBI) [29], neonatal encephalopathy [30], and emotional dysregulation [31]. Additionally, J147 exerts no significant cytotoxicity in vitro and in vivo [22, 24]. A summary of research on the therapeutic effects of J147 is detailed in Table 1. In this narrative review, we provide an overview of the activities of J147 in cells and animal models and discuss its potential mechanisms and prospects as a therapeutic agent.

**Table 1:** Summary of studies on J147

Author	J147 dose	Time after J147 evaluation	Cell and animal models		Mice age	Evaluated task	Results	Mechanisms	Disease or effect
Chen et al. 2011 [14]	1, 2, 5 mg/kg	Fed for 7 days	Sprague-Dawley Rat		7 weeks	NOR	better performance	Reduces soluble Aβ levels, oxidative stress, heat-shock proteins, and inflammation, increases synaptic protein and BDNF expression	AD
	10–20 mg/kg/day	Fed for 2 weeks	Mice		6 weeks	Barnes maze, Y-maze and NOL	better performance		
	0–20 µM	24 and 48 h	HT22, PC12, and primary cortical neurons		/	/	/		
Prior et al. 2013 [24]	10 mg/kg/day	Fed for 3 months	Mice		20 months	Two-day water maze, EPM, fear-conditioning assay, and Y maze	better performance	Rescues short-term and spatial memory	AD
	100 nM and 10 µM	≥ 1 h	HT22 and PC12		/	/	/		
Lapchak et al. 2013 [29]	1-300 µM	6 or 24 h	H4IIE cell line		/	/	/	Effects on cellular toxicity	CeeToxTM safety and genotoxicity analysis
	1-300 µM	Fed for 4 days	Rat		/	/	/		
Currais et al. 2015 [18]	10 mg/kg/day	Fed for 7 months	Mice		3–13 months	OFT, EPM, Barnes maze and object recognition	better performance	Reduces age-related RNA expression, rescues metabolomic of plasma and cortex biological pathways, decreases inflammation, and prevents alterations in Aβ and tau homeostasis	Aging and dementia
Kim et al. 2015 [32]	100 µM	7–48 h	SH-SY5Y		/	/	/	Inhibits oligomerization and fibrillation of β-amyloid peptides and protects neuronal cells from β-amyloid-induces cytotoxicity	Cytotoxicity
Chen et al. 2015 [33]	20 mg/kg	/	Mice		5–6 months	Y-maze	better performance	Induces the expression of many neurotrophic factors	AD
	0.001 to 10 µM	24 h	Primary cerebellar granule and cortical neurons, PC12		/	/	/		
Prior et al. 2016 [34]	200 ppm	Fed for 6 months	Mice		8–24 months	EPM, RI, fear conditioning, and pattern separation tests	better performance	Enhances memory, improves synaptic spine density, and stimulates neural stem and progenitor cell expansion	AD
	/	/	HT22 and MC65		/	/	/		
Daugherty et al. 2017 [35]	10 mg/kg/day	Fed for 3 months	Sprague–Dawley rat		13-months	Two-day water maze, EPM, fear-conditioning assay, and OFT	better performance	Reduces inflammation, increases for neurogenesis and synapses, and modulates fatty acid metabolism	Fatty liver disease
	10 mg/kg/day	Fed for 3 months	Mice		13 months	Two-day water maze, EPM, fear-conditioning assay, and OFT	better performance		
	6–74 nM	/	HT22, MC65, and primary cortical neurons		/	/	/		
Daugherty et al. 2018 [27]	10 mg/kg or 50 mg/kg	24 h	Mice and rats		> 12 weeks	Paw thermal sensitivity, MNCV, Rotarod and Von Frey filaments	better performance	Anti-inflammatory and activates AMP kinase pathway	Diabetes
Goldberg et al. 2018 [25]	10 mg/kg/day	Fed for 6 months	Mice		3–10 months	/	/	Increases in intracellular calcium leading to sustained CAMKK2-dependent activation of the AMPK/mTOR pathway	Aging and dementia
	0.1 or 2 µM	Fed for 10 days	Drosophila		1 week	/	extends lifespan		
	0-1000 µM	0–48 h	HT22, MC65 and primary cortical neurons		15 min	/	/		
Lian et al. 2018 [36]	2, 5 and 10 mg/kg	> 1 h	Mice		Adult	TST, FST and LAT	better performance	Increases pCREB, cAMP, PKA, and BDNF levels	Depression
Lv et al. 2018 [21]	10 mg/kg/day and 100 mg/kg/day	Fed for 5 days	Rat		Adult	Von Frey filaments	better performance	Increases AMPK expression, reduces TRPA1 expression and calcium reaction level	Diabetes
	10 µM and 100 µM	24 h	RSC96 cells		/	/	/		
Currais et al. 2019 [18]	10 mg/kg/day	Fed for 4 months	Mice		9 months & and 13 months	EPM and Barnes maze reversal	better performance	Preserves key brain mitochondrial metabolites and elevating acetyl-CoA levels reduces aspects of brain aging	Aging
	1µM, 50 and 100 nM	24 h	HT22 and primary neurons		/	/	/		
Li et al. 2020 [31]	1, 3 and 9 mg/kg	Fed for 3 days	Mice		Adult	TST and FST	better performance	Modulates 5-HT1A-dependent cAMP/PKA/pCREB/BDNF	Depression
Goldberg et al. 2020 [37]	10 mg/kg/day	Fed for 4 months	Mice	9 months and 13 months		/	/	Modulates Ca2 + metabolism and against age-related neurotoxicity	AD
	1 µM	Overnight	HT22	/		/	/		
Pan et al. 2021 [38]	10 mg/kg	Fed for 3 days	Mice	Adult		OFT, TST, FST, NSFT, SPT and LAT	better performance	Inhibits MAO-A activity and increases synaptic monoamines	Depression and anxiety
Kepchia et al. 2021 [39]	10 mg/kg/day	Fed for 4 months	Mice	9 months and 13 months		EPM	better performance	Prevent age-associated disease in brain and kidney	AD
	2 µM	1–71 days	Drosophila	1–71 days		/	extends lifespan		
Lv et al. 2021 [22]	1, 2 and 4 µM	35–60 h	Zebrafish	/		/	/	ERK pathway, anti-melanosome effects, inhibits melanin production, prevents dendrite extension and melanosome distribution	Skin-whitening
	1%	Twice a day for 3 weeks	Guinea pigs	6 weeks		/	/		
	1–8 µM	0–48 h	B16F10 murine melanocytes	/		/	/		
Kepchia et al. 2022 [19]	/	Fed for 4 months	Mice and Wistar rats	9 months and 13		/	/	Activation of the AMPK/ACC1 pathway in the liver and decreased plasma free fatty acid levels	Fatty liver disease
	1 µM	24 h	HepG2 cell	/		/	/		
Jin et al. 2022 [28]	1,10 and 30 mg/kg	72 h	Rat	Adult		/	better performance	Reduces tPA-induced brain hemorrhage	Stroke

Abbreviations:

NOR, novel object recognition. A, amyloid. BDNF, brain-derived neurotrophic factor. AD, Alzheimer’s disease. NOL, novel object location. EPM, elevated plus maze. OFT, open field test. RI, recognition index. MNCV, motor nerve conduction velocity. AMP, Adenosine monophosphate. CAMKK2, calcium/calmodulin-dependent protein kinase kinase. AMPK, AMP activated protein kinase. mTOR, mechanistic target of rapamycin. TST, tail-suspension test. FST, forced swim test. LAT, locomotor activity test. pCREB, phosphorylated CREB. CREB, cAMP-response element binding protein. cAMP, 3’, 5’-cyclic adenosine monophosphate. PKA, protein kinases A. TRPA1, ransient receptor potential A1. NSFT, novelty suppressed feeding test. SPT, sucrose preference test. MAO, monoamine oxidase. ERK, extracellular signal-regulated kinase. Acc1, acetyl-CoA carboxylase 1.

### Multifaceted therapeutic implications of J147: from molecules to diseases

#### 1. J147 reduces AD-related memory and recognition impairment

Age is known to be a risk factor for dementia, but the molecular relationship between aging and dementia remains only partly understood [40]. While half of dementia patients present with AD, treatment of AD remains challenging due to the lack of knowledge about AD pathogenesis [41]. It is conventionally believed that, along with the aging process of neurons, aggregates of amyloid beta (Aβ) and tau proteins begin to accumulate in certain regions of the brain and exhibit cytotoxicity against neurons, which leads to neuron death and, subsequently, long-term memory and cognition impair [42]. However, emerging evidence suggests that the presence of the aggregates may not be fully responsible for AD development and progression; instead, the aggregates’ deposition may result from neuron damage instead of causing it [43, 44]. Multiple other mechanisms, such as neuroinflammation, may be involved in this process and jointly regulate AD progression [45]. Due to the limited understanding of the molecular mechanisms of AD, few drugs have been approved for AD treatment, especially small-molecule compounds [46]. In particular, many drugs designed for treating AD show activities in vitro or in animal experiments but only display temporary memory and cognition enhancement in clinical trials, with little or no effect on AD progression [47]. This may be because these drugs are only effective in clearing the aggregates but fail to eliminate the actual cause of AD, which is currently unclear. The blood-brain barrier may also hinder the effectiveness of AD drugs [48].

J147 has shown promising protective activity *in* AD-related cell lines and significant memory and recognition-promoting effects in animal models [9, 14, 20, 24]. As previously noted, this may be attributed to the inhibitory activity of J147 on ATP5A, the latter of which activates the AMPK/mTOR pathway that plays a key role in aging and dementia [25]. Additionally, J147 may induce the expression of BDNF and nerve growth factor (NGF) to ameliorate neuronal damage [24]. The high bioavailability and blood-brain barrier penetration of J147 may also have contributed to its substantial therapeutic effect [23, 24]. Notably, no significant side-effects of J147 has been reported. Due to these merits, J147 has entered the Phase 1 clinical trial to assess its safety and efficacy in treating AD (ClinicalTrials.gov Identifier: NCT03838185). This trial has been completed however at the point of writing the results are not yet released.

#### 2. J147 alleviates painful symptoms of diabetic neuropathy

Diabetes is a chronic disease characterized by elevated, poorly-controlled blood glucose levels (hyperglycaemia) [49]. Diabetes can lead to severe damage of nerves and blood vessels, resulting in diabetic neuropathy, with symptoms ranging from painful stabbing or burning sensations, or tingling and numbness in the affected limbs [50]. Diabetic neuropathy threatens the health of over 50% of diabetes patients and may lead to serious infection and disability [51]. The neuroprotective activity and painful symptoms alleviation of J147 in diabetic neuropathy has been investigated in recent studies [21, 27]. Daugherty et al. found that J147 effectively reduced the levels of neuroinflammation markers, including tumor necrosis factor α (TNFα), translocator protein (TSPO), inducible nitric oxide synase (iNOS), and glial fibrillary acidic protein (GFAP), in streptozotocin (STZ)-induced diabetic mice models, possibly through activating the AMPK pathway [27]. Behavioral experiments showed that J147 treatment could reverse the diabetes-induced decreased motor nerve conduction velocity (MNCV), whereas no significant difference was observed between normal mice treated with J147 or vehicle [27]. J147 treatment could also rapidly alleviate the tactile allodynia in STZ-induced diabetic mice and rats [27]. Another study by Lv et al. revealed that J147 could reduce the mechanical withdrawal threshold (MWT) in STZ-induced diabetic rat models [21]. In vitro experiments showed that J147 could enhance the expression of AMPK, which suppresses transient receptor potential A1 (TRPA1), an ion channel responsible for sensory neural responses to mechanical and temperature stimulation in RSC96 cells [21]. This may partly explain the reduced MWT in diabetic rat models, because blocking TRPA1 could attenuate the mechanical hypersensitivity in diabetic animals [52]. The two studies suggest that J147 may be a potential drug for alleviating painful symptoms of diabetic neuropathy without introducing significant side effects.

#### 3. J147 improves tissue-type plasminogen activator treatment in ischemic stroke

Ischemic stroke, a life-threatening emergency caused by cerebral vascular blockage, accounts for 87% of all stroke events and is associated with a high disability rate, high morbidity, and high mortality [53, 54]. Recombinant tissue-type plasminogen activator (t-PA) is currently the only Federal Drug Administration (FDA)-approved drug for treating acute ischemic stroke via intravenous administration [55]. However, the time window for using t-PA is limited to 3-4.5 h from stroke onset, because a delayed t-PA treatment may increase the risk of intracranial hemorrhage [56]. Up to now, few drugs have been found effective and specific for cerebroprotection in acute ischemic stroke. Jin et al. reported that J147, in combination with t-PA at 4 h after stroke onset, could significantly reduce the infarct volume and neurological deficits in rat models with embolic middle cerebral artery occlusion [28]. Such combination treatment also alleviated the hemorrhage caused by the delayed t-PA treatment. Specifically, the administration of J147 could inhibit matrix metalloproteinase-9 (MMP-9), 15-lipoxygenase-1, and plasminogen activator inhibitor (PAI), which are key proteins that mediate the hemorrhagic transformation, neuroinflammation, and secondary microvascular thrombosis, respectively. Moreover, the combined treatment could suppress platelet activation and platelet-leukocyte aggregation in the infarct area. These results provide preliminary evidence to suggest that J147 may create a prolonged time window for t-PA use and lower the risk of hemorrhage and thus significantly reduce the mortality associated with ischemic stroke. However, more studies are required to elucidate the underlying mechanisms; also, given the complexity and heterogeineity of ischemic stroke [57], the effectiveness of J147 in different subtypes remains to be explored.

#### 4. J147 exerts antidepressant- and anxiolytic-like activity

Depression is emerging as a major public health concern worldwide: over 322 million people suffer from depression, the incidence of which is still rising [58]. Anxiety, on the other hand, affects up to 33.7% of the population at least once during the whole lifetime [59]. Traditional antidepressants and anxiolytics are effective but often accompanied by various side effects, such as dizziness, insomnia, and mental agitation [60]. Pan et al. reported that J147 inhibited MAO-A activity and increased synaptic monoamines to ameliorate both depression and anxiety-like behaviors in ICR mice [38]. Specifically, J147 could reduce the immobility time in forced swim test (FST) and tail suspension test (TST) in a dose-dependent manner, showing J147’s potential antidepressant activity [38]. On the other hand, the open field test (OFT) showed that J147 treatment could significantly increase the time spent in the central area and the number of times the center was crossed, suggesting anxiolytic effects [38]. J147 could also increase the levels of serotonin (5-hydroxytryptamine, 5-HT), a neurotransmitter seen to be downregulated in depressed patients [38]. Prior et al. also demonstrated that J147 could relieve the anxiety-like exploratory behavior in aged mice using the elevated plus maze (EPM) experiment [34]. In this study, APPswe/PS1DE9 transgenic mice were used to investigate age-related behavioral changes, including anxiety. In the EPM experiment, aged mice (24-month-old) were shown to explore significantly less compared with young mice (8-month-old). However, after switching to a diet enriched with J147 for 6 months, aged mice spent significantly more time in the EPM open arms and demonstrated increased exploring activity, interpreted as reduced levels of anxiety [34]. These findings collectively suggest that J147 may have antidepressant or anxiolytic effects in rodents with no demonstrable side-effects.

#### 5. J147 promotes whitening by suppressing melanin formation and melanosome transport

Curcumin and its derivatives (chemically modified curcumin, CMC) are potential whitening agents, as their inhibitory effects on melanogenesis have been observed in vitro [61]. Curcumin and CMCs could suppress the synthesis of melanin by inhibiting the activity of tyrosinase, as well as interfering with the uptake of melanin by keratinocytes to reduce pigmentation [61]. As a curcumin derivative with superior bioavailability and stability, J147 also exhibits hypopigmentary effects on melanocytes: Lv et al. showed that J147 could suppress both basal and α-MSH-induced melanogenesis and reduce melanocyte dendricity extension and melanosome transport [22]. Specifically, J147 could activate the extracellular signal-regulated protein kinase (ERK) pathway to induce microphthalmia-associated transcription factor (MITF) degradation, which ultimately inhibited melanin synthesis and melanosome transport [22]. The hypopigmentary effect of J147 was also validated in vivo using animal models, including zebrafish and brown guinea pigs without significant toxicity [22]. Given that only one study revealed hypopigmentary effect of J147, more investigation is required to confirm its potential in becoming a skin whitening agent in the treatment of skin pigmentation disorders.

#### *6.* J147 reduces liver and plasma free fatty acid levels

The effect of J147 on free fatty acid (FFA) levels was first noticed in the large-scale analysis of metabolites in the plasma of SAMP8 mice treated with J147: the increase in FFA levels with age could be inhibited by J147 treatment [18, 20]. These findings were later validated by Devin et al. in 2022 [19]. It was found that J147 could activate the AMPK pathway in the liver, which in turn inhibited the downstream target ACC1 via phosphorylation. ACC1 is the enzyme responsible for the conversion of acetyl-CoA into malonyl-CoA, the precursor of FFA. This finding provides support for the purported neuroprotective effects of J147 from the perspective of fatty acid metabolism, and suggests the potential role of J147 in the treatment of fatty liver diseases.

## Conclusion and outlook

In this narrative review, we provided a comprehensive overview of current research on the curcumin derivative J147, and discuss findings related to its neuroprotective and neurotrophy effects. Specifically, the studies discussed suggests a potential role for J147 in the treatment of dementia, diabetic neuropathy, ischemic stroke (in combination with t-PA), depression, and anxiety that warrants further investigation. The therapeutic effects of J147 appear to mainly be associated with its inhibition of ATP5A, which leads to an increase in cytosolic Ca^2+^ that activates the AMPK/mTOR pathway, and which plays a central role in neuronal aging and death. In addition, J147 may also serve as a whitening agent that prevents melanogenesis through the activation of the ERK pathway that leads to MITF degradation, which inhibits both melanin synthesis and melanosome transport. J147 is also a potential drug for fatty liver disease because it activates the AMPK pathway in the liver to inhibit ACC1 activity, which in turn reduces FFA synthesis to decrease plasma and liver FFA levels. Moreover, J147 has shown promising bioavailability and safety profiles in preclinical in vitro and i*n vivo* studies, suggesting potential for further investigation into its use as an orally administered treatment in various conditions. However, more extensive clinical trials are necessary to fully establish its safety and efficacy.

There are several limitations with the current studies on J147 reviewed herein. Firstly, most studies on J147 are basic or preclinical, while no clinical data is available on the therapeutic effects of J147 in humans, since the results of the only registered clinical trial have not yet been published. This has limited our understanding of its safety and efficacy beyond basic and preclinical models. Secondly, despite its diverse effects on different signalling pathways, there is only one identified molecular target of J147, ATP5A. Since ATP5A is broadly distributed in different tissues and organs, whereas J147 mainly shows its neuroprotective functions in the central or peripheral nervous systems, there might be other molecules or underlying mechanisms targeted by J147. More studies are required to elucidate the selectivity and specificity of J147. For instance, bioinformatics databases and tools, such as the ChEMBL [62], MDDR [63], and SuperPred [64] databases that store numerous protein structures and molecular fingerprints, as well as SwissTargetPrediction [65], an online web tool that predicts protein targets of certain small molecules, may be of great help identifying the potential targets of J147 *in silico*. Thirdly, due to the lack of understanding of the molecular targets of J147, little is known about its interaction mechanisms. It is still unclear whether J147 binds to its targets through covalent bonds or other interactions, such as hydrogen bonds or hydrophobic interactions. Molecular docking and molecule pull-down experiments may provide some insights into this question.

On top of its apparently broad neuroprotective functions, the novel therapeutic effects of J147 in other diseases are yet to be explored. For example, J147 may be effective in treating *sepsis-associated encephalopathy* (SAE), a serious brain dysfunction caused by infection of the body that threatens 8–70% of patients admitted to the intensive care unit [66, 67]. This is because the pathogenesis of SAE is mainly mediated by the activation of microglia [68], which may be inhibited by J147 as indicated by the stroke rat models. The inhibition of microglia activation by J147 may relieve the inflammation in the brain threatened by SAE and hence protect the neurons [14, 20]. Similarly, J147 may exert neuroprotective and neurotrophic activities in many other brain diseases involving neuroinflammation, such as multiple sclerosis and adrenoleukodystrophy [69]. While J147 has advanced to clinical trial stage for AD treatment, its potential impact on neurodegenerative diseases and other neurological conditions, such as SAE, is still under investigation. This ongoing research, including potential developments around J147 derivatives, could potentially offer novel therapeutic approaches for these conditions. However, it is crucial to underscore that these are early-stage findings and further rigorous studies are required to fully validate these prospects.

## Data Availability

N/A.

## Abbreviations

5-HT: 5-hydroxytryptamine
Aβ: Amyloid beta
ACC1: Acetyl-CoA carboxylase 1
AD: Alzheimer’s disease
AMP: Adenosine monophosphate
AMPK: AMP activated protein kinase
ATP5A: α-F1-ATP synthase
BACE: β-secretase
BDNF: Brain-derived neurotrophic factor
CAMKK2: Calcium/calmodulin-dependent protein kinase kinase β
cAMP: 3’, 5’-cyclic adenosine monophosphate
cLogP: Partition coefficient
CMC: Chemically modified curcumin
CNS: Central nervous system
CREB: cAMP-response element binding protein
EPM: Elevated plus maze
ERK: Extracellular signal-regulated protein
FDA: Federal Drug Administration
FFA: Free fatty acid
FST: Forced swimming test
GFAP: Glial fibrillary acidic protein
iNOS: Inducible nitric oxide synase
IS: Ischemic stroke
LAT: Locomotor activity test
MAO: Monoamine oxidase
MITF: Microphthalmia-associated transcription factor
MMP-9: Matrix metalloproteinase-9
MNCV: Motor nerve conduction velocity
mTOR: Mechanistic target of rapamycin
NGF: Nerve growth factor
NOL: Novel object location
NOR: Novel object recognition
NSFT: Novelty suppressed feeding test
OFT: Open field test
PAI: Plasminogen activator inhibitor
PKA: Protein kinase A
pCREB: Phosphorylated CREB (cAMP-response element binding protein)
RI: Recognition index
RO5: Lipinski’s rule of five
SAE: Sepsis-associated encephalopathy
SAMP8: Senescence-accelerated prone 8
SD: Sprague dawley
SPT: Sucrose preference test
STZ: Streptozotocin
TBI: Traumatic brain injury
TNFα: Tumor necrosis factor Alpha
t-PA: Tissue-type plasminogen activator
TRPA1: Ransient receptor potential A1
TSPO: Translocator protein
TST: Tail suspension test
